# Variability of Phenolic Compound Accumulation and Antioxidant Activity in Wild Plants of Some *Rumex* Species (*Polygonaceae*)

**DOI:** 10.3390/antiox11020311

**Published:** 2022-02-03

**Authors:** Pavel Feduraev, Liubov Skrypnik, Sofia Nebreeva, Georgii Dzhobadze, Anna Vatagina, Evgeniia Kalinina, Artem Pungin, Pavel Maslennikov, Anastasiia Riabova, Olesya Krol, Galina Chupakhina

**Affiliations:** Institute of Living Systems, Immanuel Kant Baltic Federal University, 236040 Kaliningrad, Russia; lskrypnik@kantiana.ru (L.S.); snebreeva@stud.kantiana.ru (S.N.); gdzhobadze@stud.kantiana.ru (G.D.); avatagina@stud.kantiana.ru (A.V.); eakalinina1@kantiana.ru (E.K.); apungin@kantiana.ru (A.P.); pmaslennikov@kantiana.ru (P.M.); avryabova@stud.kantiana.ru (A.R.); okrol@kantiana.ru (O.K.); gchupakhina@kantiana.ru (G.C.)

**Keywords:** sorrel, dock, ethnobotany, medicinal plants, edible plants, chemotaxonomy, phylogeny, secondary metabolites, superfood, raw material

## Abstract

Today, more than ever, the search for non-trivial sources of biologically active substances is critical. Plants of the genus *Rumex* are noteworthy. Plants of this genus stand out for a number of advantages from the dominant plant core of meadow phytocenoses of the temperate climatic zone: a short growing season, an intensive increase in biomass, and undemanding growth conditions. In addition, this plant genus is known as a super-producer of secondary phenolic compounds. The wide distribution and intensive synthesis of biologically active substances make plants from the genus *Rumex* a promising object for study. Seven species of the genus *Rumex* (*R*. *acetosa*, *R. acetosella*, *R. confertus*, *R. crispus*, *R. maritimus*, *R.* *obtusifolius*, and *R. sanguineus*) were analyzed. Plants were collected under relatively uniform growing conditions. For subsequent extraction and analysis of phenolic compounds, as well as antioxidant activity, plants leaves were used. *R. acetosella*, *R. crispus*, *R. maritimus*, *R. obtusifolius*, and *R. sanguineus* were characterized by a high total content of phenolic compounds (111–131 mg g^–1^). The maximum content of flavonoids was found in the leaves of *R. maritimus* and *R. acetosella*. At the same time, according to high-performance liquid chromatography with diode-array detection (HPLC-DAD) analysis, derivatives of flavones (apigenin and luteolin) predominated in the leaves of *R. acetosella*, while in other species, mainly derivatives of flavonols (quercetin and kaempferol) were identified. Plants of *R. acetosa*, in comparison with other studied species, were characterized by a lower content of the studied groups of phenolic compounds, with the exception of hydroxycinnamic acids, the content of which in this species was comparable to the content of flavonoids. The maximum content of catechins was found in *R. sanguineus*; proanthocyanidins—in *R. sanguineus*, *R. obtusifolius*, and *R. crispus*; and tannins—in *R. obtusifolius*. Extracts from *R. crispus* were characterized by high antioxidant activity, measured by 2,2-diphenyl-1-picrylhydrazyl (DPPH), 2,2′-azino-bis(3-ethylbenzothiazoline-6-sulfonic acid (ABTS), and ferric reducing antioxidant power (FRAP) assays. In addition, the assessment of the phenolic profile of the plant made it possible to group the plants within the framework of cluster analysis. The distribution pattern in the clusters corresponded to the generally accepted taxonomy, with a characteristic division into subgenera (*Acetosa*, *Acetosella*, and *Rumex*). Thus, the phenolic profile can be considered as an additional instrumental approach when drawing up a systematic hierarchy.

## 1. Introduction

In Europe, the consumption of wild edible plants has been an integral part of human nutrition and traditional medicine since ancient times [1,2]. However, despite the long history of research on wild-growing plants, scientific interest in them has not weakened for multiple reasons. First, edible wild plants are known to be a good source of primary nutritional compounds (proteins, fats, sugars, vitamins, and minerals) [3]. Second, edible wild plants contain various biologically active components that demonstrated health benefits effects (flavonoids, phenolic acids, anthocyanins, tannins, terpenoids, steroidal saponins, glucosinolates, and so on) [2]. This shows their potential as nutritional supplements, feed additives, and medicinal agents [2,4]. Third, wild plants provide a colossal genetic resource that can be used in breeding programs to increase the resistance of cultivated plants and to improve their nutritional and pharmacological value [5].

Among wild plants, *Rumex* plants have a great potential. They are already widely used as food, fodder, melliferous, and medicinal plants [6,7,8]. The *Rumex* L. genus, from the *Polygonaceae* Juss. family, has about 200 species. Plants of the *Rumex* genus are common in Europe, Asia, Africa, and North America, but more widely spread in the temperate zone of the northern hemisphere [7].

In some regions, the leaves of Rumex plants (such as *R. acetosa*, *R. acetosella*, *R. abyssinicus*, *R. crispus*, *R. induratus*, *R. obtusifolius*, *R. sanguineus*, *R. tuberosus*, *R. thyrsiflorus*, and *R. vesicarius*) are used for food, mainly as salads [7,9]. The consumption of the Rumex species can be restricted owing to large amounts of oxalic acid and hydroxyanthracene derivatives present, which can cause serious health problems when consumed in high doses [9]. However, the latter accumulate mainly in the roots of Rumex plants, and not in the leaves [10].

Several *Rumex* species are included in the pharmacopoeias of various countries. For example, *R. crispus* is listed in the American Herbal Pharmacopoeia as a general detoxifier and an agent for skin treatment [11]. The State Pharmacopoeia of the Russian Federation includes the roots of *R. confertus* as a herbal medicine, which is used in the treatment of liver diseases, dysentery, pulmonary, and uterine bleeding, as well as a laxative [12]. In Nigerian, Indian, Chinese, and Indonesian medicine, the leaves of *R. nepalensis* are traditionally used for their diuretic, astringent, laxative, and sedative properties [13].

Plants of the *Rumex* genus are rich in secondary metabolites, in particular phenylpropanoids and anthraquinones, which are likely to be responsible for the medicinal properties attributed to these species [14]. The list of anthraquinones particularly common in *Rumex* plants includes, but is not limited to, chrysophanol, physcion, emodin and their glycosides, rhein, nepodin, and so on [10]. Despite the possible toxic effect mentioned above, these compounds also show anticarcinogenic, anti-inflammatory, antiarthritic, antifungal, antibacterial, antioxidant, and diuretic activity [15,16]. Flavonoids are another important class of compounds that determine the therapeutic effect of *Rumex* plants. Derivatives of kaempferol, quercetin, apigenin, luteolin, and catechins, as well as derivatives of benzoic and cinnamic acids, lignans, coumarins, and proanthocyanidins, have been isolated from various *Rumex* species [17]. Phenolic compounds are known to have strong antioxidant as well as cardioprotective, immune system promoting, antibacterial, anti-cancer, and anti-inflammatory effects [18].

According to the number of publications presented in the review of *Rumex* species [8], *R. acetosa*, *R. obtusifolius*, *R. crispus*, *R. acetosella*, and *R. dentatus* are studied the most. However, the available data on the comparison of the phytochemical composition of *Rumex* plants growing in the same territory are exceedingly rare. The aim of this study is the comparative analysis of the quantitative and qualitative composition of phenolic components, as well as the antioxidant activity of extracts of seven *Rumex* species (*R. acetosa*, *R. acetosella*, *R. confertus*, *R. crispus*, *R. maritimus*, *R. obtusifolius*, and *R. sanguineus*), growing in similar environmental conditions. The results of the study will allow not only to identify the most promising species for pharmaceutical and food use, but also to demonstrate the possibility of using their phenolic composition as an additional tool for systematizing species of the *Rumex* genus.

## 2. Materials and Methods

### 2.1. Plant Material

Plant leaves of the following species were used as the objects of study: *R. acetosa* L., *R. acetosella* L., *R. confertus* Willd., *R. crispus* L., *R. maritimus* L., *R. obtusifolius* L., and *R. sanguineus* L. The collection of plants was carried out in July 2021 in Svetlogorsk (Kaliningrad region), which is characterized by a low anthropogenic load, the absence of near major highways, industrial production, and agricultural fields. All experimental plants were harvested in the flowering phase. Species were identified by PhD A. Pungin. Voucher specimens were deposited in the herbarium of Immanuel Kant Baltic Federal University (KLGU Herbarium).

Leaf samples (4–5 leaves per plant) were taken from the top of 3–5 plants of each species. In the laboratory, leaves were washed and dried at 60 °C to constant weight. The dried leaves were crushed to a particle size passing through a 1 mm sieve. All leaves from plants of the same species constituted a combined sample, which was used to further analyze the phenolic composition and antioxidant activity.

### 2.2. Extract Preparation

Phenolic compounds were extracted from ground dry plant material using a 70% ethanol solution. A sample of the plant material of 1 g was placed in a round bottom flask containing about 40 mL of 70% ethanol, then heated in a water bath at 60 °C under reflux for 1 h. The mixture was then filtered into a volumetric flask. The extraction procedure was repeated three times. The resulting filtrate fluids were combined and brought to 100 mL with 70% ethanol solution.

### 2.3. Determination of Total Contents of Some Groups of Phenolic Compounds

#### 2.3.1. Determination of Total Phenolic Content

Spectrophotometric analysis with the Folin–Ciocalteu reagent was performed to assess the content of phenolic compounds [19]. Briefly, 2.5 mL of plant extract obtained as described above or standard solution was mixed with 1.25 mL 0.2 M Folin–Ciocalteu reagent, placed in darkness, and incubated for 10 min at room temperature. Then, 1.25 mL of 7.5% sodium carbonate solution was added to the mixture and the reaction mixture was incubated for 30 min at room temperature. The absorbance of the solutions was measured at 765 nm using a UV-3600 spectrophotometer (Shimadzu, Kyoto, Japan). Gallic acid was used as a standard. The total phenolic content (TPC) was assessed using the calibration curve and expressed in mg of gallic acid equivalents per gram of dry weight (mg GAE g^–1^ DW).

#### 2.3.2. Determination of Total Flavonoid Content

Complexation with aluminum chloride in the presence of sodium nitrite in an alkaline medium was carried out to assess the content of flavonoids, according to Sevket et al. [20]. Briefly, 100 μL of plant extract or standard solution was mixed with 300 μL of 5% sodium nitrite solution and incubated for 5 min. Then, 300 μL of 10% aluminum chloride solution was added to the mixture and the reaction mixture was incubated for 6 min. Further, 2 mL of 1 M NaOH was added, and the mixture was brought to 10 mL by distilled water. The absorbance of the solutions was measured at 510 nm using a UV-3600 spectrophotometer (Shimadzu, Kyoto, Japan). Rutin was used as a calibration standard. The total flavonoids content (TFC) was expressed in mg of rutin equivalents per gram of dry weight (mg RE g^–1^ DW).

#### 2.3.3. Determination of Total Content of Hydroxycinnamic Acids

The total content of hydroxycinnamic acids was assessed based on the reaction with Arno’s reagent, according to Štefan et al. [21]. The reaction mixture consisted of 1 mL of plant extract or standard solution, 2 mL of 0.5 M HCl, 2 mL of Arno’s reagent obtained by blending sodium nitrite and sodium molybdate (at the ratio 1:1), and 2 mL of 8.5% NaOH. The entire volume of the solution was adjusted to 10 mL by distilled water. The absorbance of the solutions was measured at 505 nm using a UV-3600 spectrophotometer (Shimadzu, Kyoto, Japan). Chlorogenic acid was used as a calibration standard. The total content of hydroxycinnamic acids (THA) was assessed using the calibration curve and expressed in mg of chlorogenic acid equivalents per gram of dry weight (mg CAE g^–1^ DW).

#### 2.3.4. Determination of Total Content of Proanthocyanidins

Butanol-hydrochloric acid reagent containing iron (II) sulfate was used to determine the amount of proanthocyanidins, according to Chupin et al. [22]. The reaction mixture consisted of 9 mL of acidified butanol containing iron sulfate (77 mg FeSO_4_ × 7H_2_O in 500 mL HCl/BuOH (2/3)) and 1 mL of plant extract. The reaction mixture was incubated in a water bath at 95 °C for 30 min. The absorbance of the solutions was measured at 520 nm using a UV-3600 spectrophotometer (Shimadzu, Kyoto, Japan). The total content of proanthocyanidins (PAs) was expressed in mg of cyanidin equivalents per gram of dry weight (mg CyE g^–1^).

#### 2.3.5. Determination of Total Catechin Content

The catechin content was determined spectrophotometrically using a vanillin reagent, according to He et al. [23]. Briefly, 1 mL of plant extract or standard solution was mixed with 4 mL of vanillin reagent (1% solution of vanillin in concentrated HCl). The blank solution was used a mixture of plant extract (or standard) and concentrated HCl. The reaction mixture was incubated for 5 min at room temperature. The absorbance of the solutions was measured at 520 nm using a UV-3600 spectrophotometer (Shimadzu, Kyoto, Japan). Standard solutions of catechin were used to plot a calibration curve. The total catechin content (TCC) was expressed in mg of catechin equivalents per gram of dry weight (mg CE g^−1^ DW).

#### 2.3.6. Determination of Total Tannin Content

The content of tannins was assessed using the Prussian blue reaction, as described earlier [24]. The analysis included two steps. First, the total content of polyphenols was determined using iron (III) chloride and potassium ferricyanide. Briefly, 250 μL of the extract or standard solution was mixed with 25 mL of distilled water, and 3 mL of a 0.5 M solution of FeCl_3_ and 3 mL of 0.008 M K_3_Fe(CN)_6_ were added. The absorbance of the solutions was measured at 720 nm after incubation for 15 min using a UV-3600 spectrophotometer (Shimadzu, Kyoto, Japan). Next, the tannins were precipitated from the extract using casein. Briefly, 0.24 g of casein was added to 10 mL of ethanol extract, and the mixture was stirred and incubated at 30 °C for 1 h. The resulting mixture was filtered, and the analysis for polyphenols was repeated with the filtrate. The difference in the results between the first and second tests was taken as the tannin content. Gallic acid was used as a standard to plot a calibration curve. The total tannin content (TTC) was expressed in mg of gallic acid equivalents per gram of dry weight (mg GAE g^–1^ DW).

### 2.4. High-Performance Liquid Chromatography with Diode-Array Detection (HPLC-DAD) Analysis of Individual Phenolic Compounds

**Figure A1:**
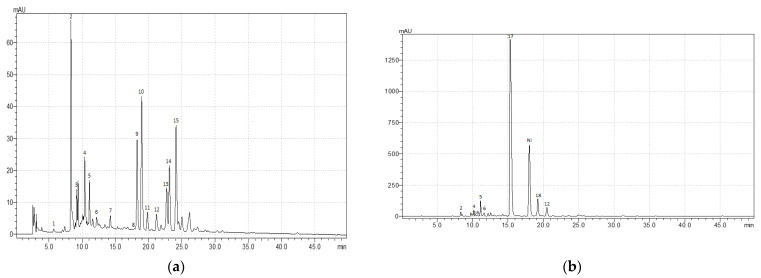

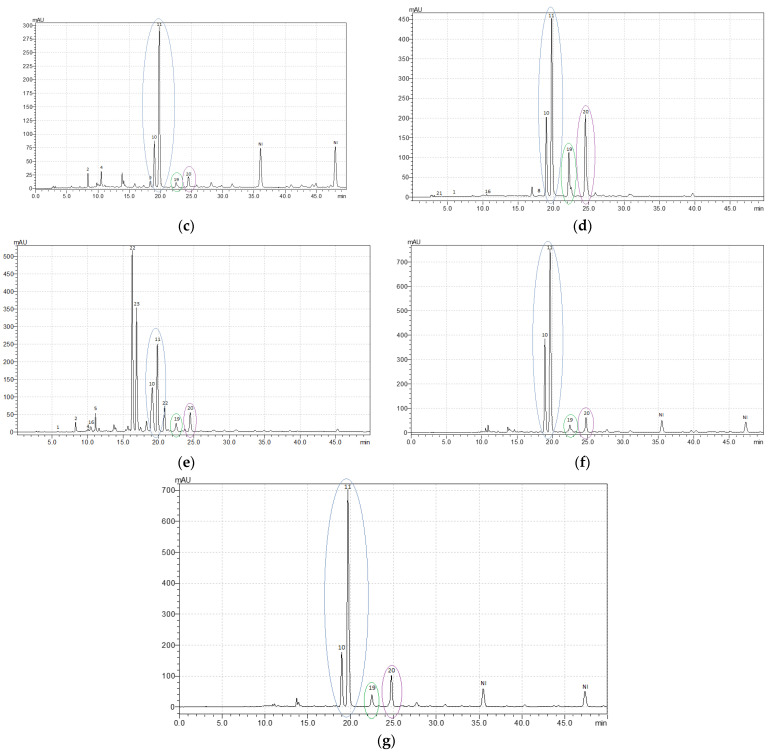
Chromatographic profile of the phenolic acids and flavonoids at 340 nm for (**a**) *R. acetosa*, (**b**) *R.acetosella*, (**c**) *R. confertus*, (**d**) *R. crispus*, (**e**) *R. maritimus*, (**f**) *R. obtusifolius*, (**g**) *R. sanguineus*: 1—protocatechuic acid; 2—sinapic acid; 3—caftaric acid; 4—chlorogenic acid; 5, 6—hydroxybenzoic acid derivatives; 7—p-coumaric acid; 8—ellagic acid; 9—quercetin derivative; 10—rutin; 11—isoquercitrin; 12 –cymaroside; 13, 14, 15—quercetin derivatives; 16—caffeic acid; 17—luteolin derivative; 18—apigenin derivative; 19—kaempferol derivative; 20—astragalin; 21—gallic acid; 22, 23—quercetin derivatives; 24—kaempferol derivative.

In preparation for HPLC analysis, the extracts obtained as described above (Section 2.2) were filtered and concentrated on a rotary evaporator, then the resulting dry matter was dissolved in 10% methanol solution. The new extract was centrifuged at 4500× *g* for 15 min, and the supernatant was filtered through a syringe filter (0.22 μm). The separation of substances was carried out on a Shimadzu LC-20 Prominence chromatograph with a Shimadzu SPD20MA diode array detector and a Phenomenex Luna column (C18 250 × 4.6 mm^2^, 5 μm). The mobile phase included a mixture of solvents: water/acetic acid 99.5/0.5 (solvent A) and acetonitrile (B). The gradient mode was used for separation: 0 min—95% A, 5% B; 3 min—88% A, 12% B; 46 min—75% A, 25% B; 49.5 min—10% A, 90% B; 52 min—10% A, 90% B; 52.7 min—95% A, 5% B; 59 min—95% A, 5% B. The flow rate was 0.85 mL min^−1^, the column temperature was 40 °C; sample volume—20 μL. Detection was carried out in the wavelength range of 180–900 nm. The exemplary HPLC chromatograms of the phenolic acids and flavonoids in different *Rumex* species are presented in Appendix A on Figure A1.

**Figure A2:**
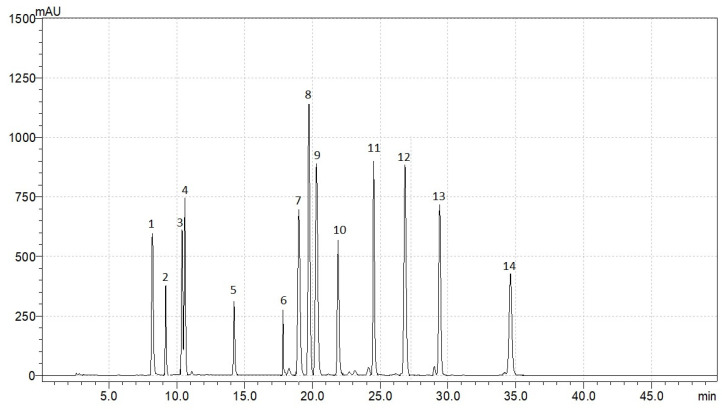
Chromatographic profile of the phenolic acids and flavonoids at 340 nm for standard mixture: 1—sinapic acid, 2—caftaric acid, 3—chlorogenic acid, 4—caffeic acid, 5—p-coumaric acid, 6—ellagic acid, 7—rutin, 8—isoquercitrin, 9—cynaroside, 10—chicoric acid, 11—astragalin, 12—apigenin 7-O-glucoside, 13—rosmarinic acid, 14—baicalin.

The compounds of interest were identified by comparing their peak retention times and UV spectra with those of the chromatographically pure samples. Chromatograms were processed using the “LabSolutions” software. Quantitative analysis of the flavonoids was carried out using calibration curves plotted in the concentration range of 10–100 μg mL^−1^. The following standards were used: caftaric acid, chicoric acid, chlorogenic acid, p-coumaric acid, rosmarinic acid, sinapic acid, trans-caffeic acid, 3,4-dihydroxybenzoic acid, gallic acid, ellagic acid, luteolin 7-O-glucoside, apigenin 7-O-glucoside, apigenin 7-O-glucuronide, quercetin 3-O-rutinoside, quercetin 3-β-D-glucoside, kaempferol 3-O-glucoside, baicalin, diosmin, and catechin. All standards were purchased from Sigma-Aldrich (Sigma-Aldrich Rus, Moscow, Russia). The chromatogram of the mixture of standards is presented in Appendix A on Figure A2.

### 2.5. Determination of Antioxidant Activity

The antioxidant activity of the extracts was assessed based on the ability to scavenge the 2,2-diphenyl-1-picrylhydrazyl (DPPH) and 2,2′-azino-bis(3-ethylbenzthiazoline-6-sulfonic acid (ABTS)) radicals, as well ferric reduced antioxidant power as the ability to reduce Fe^3+^ in the 2,4,6-tripiridyl-s-triazine complex (FRAP) [25]. Briefly, for the DPPH-assay, 30–100 μL of plant extract was added to 2.85 mL of 0.1 mM DPPH-solution. The reduction of absorbance was measured at 515 nm after 30 min incubation of the reaction mixture at room temperature in darkness. For the ABTS-assay, 2.85 mL of ABTS solution was mixed with 150 μL of plant extracts. ABTS radical was generated by mixing aliquot parts of 7.0 mM ABTS-solution and 2.45 mM potassium persulfate solution. After exactly 15 min, the absorbance of reaction mixture was measured at 734 nm. In the FRAP-assay, the reaction was started by mixing 3.0 mL of FRAP reagent with 100 μL of plant extract. The FRAP reagent was freshly prepared by mixing 10 parts of 0.3 M acetate buffer (pH 3.6), 1 part of 10 mM 2,4,6-tripyridyl-triazine (TPTZ) in 40 mM HCl, and 1 part of 20 mM FeCl_3_ × 6H_2_O. After 10 min incubation at 37 °C in darkness, the absorbance was measured at 593 nm. The absorbance in all assays was measured using a UV-3600 spectrophotometer (Shimadzu, Kyoto, Japan). As a blank solution in DPPH-, ABTS-, and FRAP-assays, a mixture containing the appropriate reagent and 70% ethanol was used instead of extract. Trolox was used as a calibration standard in all methods. Antioxidant activity was expressed in mg of Trolox equivalents per gram of dry weight (mg TE g^–1^).

### 2.6. Statistical Analysis

All experiments were carried out in triplicate. The analytical results are presented as mean ± standard deviation. To analyze the dependence of quantitative traits, Pearson’s correlation coefficient was used. Mean values of studied variables were used for correlation analysis (*n* = 7). The level of significance was established at *p* ≤ 0.05. The heat map and clusters are built based on the normalized mean values of the analyzed variables using the 2019b program (OriginLab Corporation, Northampton, MA, USA). Euclidean distance was used as a measure of similarity.

## 3. Results

### 3.1. Variation in the Content of Some Groups of Phenolic Compounds

In the phenolic composition study, *R. acetosella*, *R. crispus*, *R. maritimus*, *R. obtusifolius*, and *R. sanguineus* demonstrated the highest values (Table 1). The total phenolics content in their leaves ranged from 111 to 131 mgg^−1^. The leaves of *R. confertus* showed an even lower TPC (about 76 mg g^−1^), whereas *R. acetosa* was characterized by the lowest value (about 23 mg g^−1^).

A high content of flavonoids was characteristic of the leaves of *R. maritimus* and *R. acetosella* (Table 1). The total flavonoids content in the leaves of *R. crispus*, *R. obtusifolius*, and *R. sanguineus* varied from 92 to 98 mg g^−1^. A notably lower content of flavonoids was found in the leaves of *R. confertus* (about 38 mg g^−1^) and *R. acetosa* (about 18 mg g^−1^).

The hydroxycinnamic acids’ accumulation in the leaves of the studied species showed a somewhat different tendency (Table 1). The highest content was found in the leaves of *R. acetosella* (about 18 mg g^−1^). However, as opposed to the TPC and TFC values, the leaves of *R. acetosa* were characterized by a high total content of hydroxycinnamic acids as well (up to 13 mg g^−1^). Whereas the leaves of *R. obtusifolius* and *R. sanguineus* did not show THA values higher than 2 mg g^−1^ (Table 1).

The highest total catechins content was found in the leaves of *R. sanguineus*—about 11 mg g^−1^ (Table 1). The TCC values of *R. obtusifolius*, *R. confertus*, *R. crispus*, and *R. maritimus* leaves were almost twice as low (from 4.8 to 6 mg g^−1^). The *R. acetosa* and *R. acetosella* leaves demonstrated the lowest catechin content (from 0.9 to 1.3 mg g^−1^).

The leaves of *R. sanguineus*, *R. obtusifolius*, and *R. crispus* were shown to have a high amount of proanthocyanids (from 6.4 to 7.2 mg g^−1^). The lowest PA content was found in the leaves of *R. acetosa* (0.24 mgg^−1^) (Table 1). The leaves of *R. acetosa* were also characterized by a very low content of tannins (less than 0.5 mg g^−1^), while the highest level of TTC was found in the leaves of *R. obtusifolius* (about 17 mg g^−1^) (Table 1).

Thus, various species of *Rumex* were associated with their own maxima of individual phenolic group levels: *R. maritimus*—flavonoids, *R. acetosella*—hydroxycinnamic acids, *R. sanguineus*—catechins, *R. sanguineus*, *R. obtusifolius*, *R. crispus*—proanthocyanidins, *R. obtusifolius —*tannins. The leaves of *R. acetosa* were characterized by the lowest contents of all analyzed phenolic compounds, except for the THA level (Table 1).

### 3.2. Variation in the Content of Individual Phenolic Compounds

Despite the low value of TPC, *R. acetosa* demonstrated a remarkable diversity of phenolic compounds, especially phenolic acids (Table 2; Appendix A, Figure A1a). The leaves of *R. acetosa* contained protocatechuic acid, sinapic acid, caftaric acid, chlorogenic acid, p-coumaric acid, ellagic acid, and other hydroxybenzoic acid derivatives. Among them, sinapic acid was the most present (about 5 mg g^−1^). Moreover, multiple types of flavonoids, such as derivatives of quercetin (rutin, isoquercitrin, and so son) and luteolin (cynaroside), were found in the leaves.

A characteristic feature of *R. acetosella* was the presence of mostly flavones (derivatives of luteolin and apigenin) in the leaves, in contrast to other species, where flavonols (derivatives of quercetin and kaempferol) prevailed (Table 2; Appendix A, Figure A1b). Moreover, *R. acetosella* was characterized by a diverse composition and a high content of phenolic acids. The leaves are shown to contain protocatechuic acid, sinapic acid, chlorogenic acid, caffeic acid, and other derivatives of hydroxybenzoic acids.

The leaves of *R. confertus*, *R. crispus*, *R. maritimus*, *R. obtusifolius*, and *R. sanguineus* showed the presence of rutin and isoquercitrin, the content ratio of which varied in these species from 1:2.5 to 1:5.8, as well as the presence of astragaline and another kaempferol derivative (Table 2; Appendix A, Figure A1c–g). In fact, *R. confertus*, *R. crispus*, *R. obtusifolius*, and *R. sanguineus* demonstrated a higher level of isoquercitrin compared with other phenolic compounds present in the leaves.

The leaves of *R. crispus* were characterized by a high content of kaempferol derivatives (about 37 mg g^−1^ in total) and gallic acid (about 5 mg g^−1^) compared with other studied species (Table 2; Appendix A, Figure A1d).

The *R. maritimus* sample showed the highest concentration of quercetin derivatives (Table 2). Moreover, this species was characterized by a rich qualitative composition of phenolic acids. It includes protocatechuic acid, sinapic acid, chlorogenic acid, caffeic acid, and other derivatives of hydroxybenzoic acids.

The leaves of *R. obtusifolius* and *R. sanguineus* had a similar metabolic profile with high levels of flavonoids (quercetin derivatives) and very low levels of phenolic acids (Table 2; Appendix A, Figure A1f,g). However, it should be noted that the leaves of *R. sanguineus* were high in catechin (up to 12 mg g^−1^), in contrast to *R. obtusifolius* and other analyzed species.

### 3.3. Antioxidant Activity of the Rumex Extracts

Extracts from *R. crispus* demonstrated high antioxidant activity based on all three methods (Table 3). A high level of antioxidant activity was also found in *R. maritimus* extracts (according to the ABTS and FRAP methods). The lowest antioxidant activity was shown by the extracts of *R. acetosa* (Table 3).

### 3.4. Correlation between Phenolic Compounds Content and Antioxidant Activity

Antioxidant activity is caused by the presence of certain components in plant samples, usually compounds of phenolic nature. Correlation analysis carried out during this study proved a positive relationship between antioxidant activity and the total content of phenolic compounds (*r* = 0.785–0.921, *p* ≤ 0.05), flavonoids (*r* = 0.602–0.918, *p* ≤ 0.05), proanthocyanidins (*r* = 0.721–0.842, *p* ≤ 0.05), and tannins (*r* = 0.591–0.776, *p* ≤ 0.05) (Table 4). However, the results related to the content of hydroxycinnamic acids were unexpected. Either there was no significant correlation between the antioxidant activity level (according to the DPPH and ABTS methods) and THA, or there was an inverse correlation of moderate strength (when based on the FRAP method).

### 3.5. Heat Map and Cluster Analysis of Studied Rumex Species Based on the Content of Phenolic Compounds and Antioxidant Activity of Their Extracts

**Figure 1:**
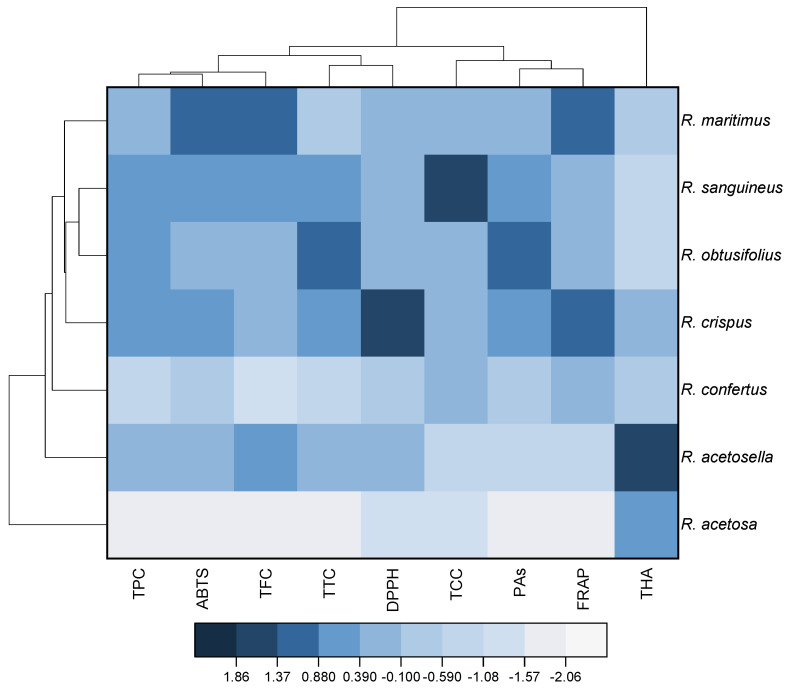
Heat map with clusters for studied variables (at the top) and *Rumex* species (at the left). TPC, total phenolics content; TFC, total flavonoids content; THA, total hydroxycinnamic acids; TCC, total catechins content; PAs, total proanthocyanidins content; TTC, total tannins content; DPPH, antioxidant activity determined by the DPPH (2,2-diphenyl-1-picrylhydrazyl) assay; ABTS, antioxidant activity determined by the ABTS (2,2′-azino-bis(3-ethylbenzothiazoline-6-sulfonic acid)) assay; FRAP, ferric reducing antioxidant power.

Based on the normalized values of the studied parameters, a heat map with cluster analysis was built (Figure 1). The dendrogram presented in Figure 1 (top) demonstrates that all the studied parameters can be divided into four clusters. The first cluster includes total phenolic content, antioxidant activity according to the ABTS method, and the total flavonoid content. The second cluster consists of total tannin content and antioxidant activity based on the DPPH method. The third cluster includes the total content of catechins, proanthocyanidins, and antioxidant activity based on the FRAP method. A separate cluster is formed by hydroxycinnamic acids.

The dendrogram on the left shows that the analyzed *Rumex* species can be divided into two large clusters (Figure 1). The first of them consists of only *R. acetosa*, and the second of all the other studied species. The second cluster includes multiple groups. One of them has only *R. acetosella*, whereas the other group includes *R. sanguineus*, *R. obtusifolius*, *R. crispus*, and *R. confertus*. The dendrogram shows that, in the latter group, *R. sanguineus* and *R. obtusifolius* in turn form a micro-group characterized by very similar composition.

## 4. Discussion

Nowadays, the role of secondary metabolites as regulatory and adaptogenic is not questioned. For instance, the wide geographical distribution of the *Rumex* plants can be partly associated with the flexible system of secondary metabolism. In this study, wild plants with relatively uniform growing conditions were used. The collection sites were located in similar climatic and landscape conditions, with a low anthropogenic load. In addition, the plants were analyzed within the same ontogenetic phase—the flowering phase. This point is fundamental, as the level of regulatory secondary compounds can differ significantly at different stages of growth [26].

### 4.1. Approaching the Problem of the Rumex Taxonomy

In accordance with the classical taxonomy, which is based on the assessment of morphological features and karyotypes, the genus *Rumex* is divided into four subgenera: *Acetosella*, *Acetosa*, *Platypodium*, and *Rumex* [27,28]. The subgenus *Acetosa* (section *Acetosa*) consists of *Rumex acetosa* and its relatives, which form a homogeneous group of species with similar morphological and karyological characteristics, including a homogeneous sex chromosome system. A distinctive feature of the subgenus *Acetosella* is heteromorphic sex chromosomes. This cytological feature allowed the subgenus *Acetosella* to be classified as a distinct taxonomic group [28]. The classification of the subgenus *Rumex*, and in particular the identification of individual subsections within this subgenus, was based not only on the cytological and morphological characteristics of the species, but also on their geographical distribution. In the context of the studied species, the following subsections of the subgenus *Rumex* should be separately mentioned: subsection *Patientiae* (*R. crispus* and *R. confertus*), subsection *Optusifolii* (*R. optusifolius* and *R. sanguineus*), and subsection *Orientalis* (*R. maritimus*) [29,30].

However, modern approaches to the taxonomy of wild species, including certain species of *Polygonaceae* Juss., are somewhat limited. Most important is the lack of materials for molecular genetic studies used to appropriately hierarchize the species. There are a small number of studies attempting to systematize plants of the family *Polygonagea*, the *Rumex* genus in particular, by comparing rather conservative regions of the chloroplast genome (such as *trnH*-*psbA*, *rbcL*, and *trnL*-*F*) or fragments of the nuclear genome (*nrITS*) [31,32]. However, the taxonomy of the *Polygonaceae* Juss. is constantly being refined. The reason for such changes is the revision of the knowledge on individual genera and/or the emergence of new phylogenetic data [31,33]. Often, these findings are contradictory, and there is a need for additional tools that can clarify the relations among the plants of the *Polygonaceae* Juss. in general, and of the *Rumex* genus in particular. Metabolic profiling can be such a tool.

Evaluation of the secondary metabolites’ profile can make some adjustments to the existing taxonomy, or vice versa—confirming the current morphoanatomical and phylogenetic data. This way, for example, the identification of smaller genera, carried out by N.N. Tsvelev in 1993, was confirmed by studying the distribution of phenolic compounds in the species *Polygonum* L. [34]. The phenolic composition study performed on several species of the genera *Aconogonon* (Meissn.) Reichenb., Bistorta Hill, and Persicaria Mill showed their taxon-specificity, as well as the potential of metabolic profiling as a taxonomic tool for plants at various levels [35]. In our study, attempts were also made to find a connection between the accumulation of various groups of phenolic compounds and specific species of the *Rumex* genus. This way, the heat map (Figure 1) demonstrates that, based on the nature of the accumulation of bioactive phenolic compounds, as well as the antioxidant activity of plants *Rumex acetosa* and *Rumex acetosella*, these species belong to two separate groups. These results are consistent with both the “classical” taxonomy and with the latest data on the division of species by specific genetic markers [36]. The *R. obtusifolius* and *R. sanguineus* species, sharing the same heatmap group and the same subsection of *Optusifolii*, provide further evidence. On the other hand, *R. confertus* and *R. crispus*, which canonically belong to the same subsection *Patientiae*, were placed in separate clusters.

The presence of specific compounds and their derivatives is another taxonomic separator. For example, in the samples of *Rumex acetosella*, derivatives of kaempferols and quercetin are not present, whereas derivatives of flavones are common. On the contrary, in the samples of *R. confertus*, *R. crispus*, *R. maritimus*, *R. obtusifolius*, and *R. sanguineus*, derivatives of flavonols (kaempferols and quercetin) are found, but derivatives of flavones are not (Table 2).

The analysis of biochemical markers should not act as a competitive approach in the formation of taxonomic groups de novo. This tool can be used to refine, adjust, and support existing systematic approaches.

### 4.2. The Rumex Plants as a Resource Object Specificity of Use

Plants of the genus *Rumex* have traditionally been used as edible or medicinal plants in various regions of the world. However, today, their biotechnological potential is becoming evident, and these species can act as a resource of biologically active substances.

The *Rumex* plants are abundant, undemanding, gain phytomass easily, and have a short vegetative cycle (and, as a consequence, can reproduce frequently throughout the year), thus they have a real advantage among wild plants of the temperate zone. It should also be noted that *Rumex* species have a high potential for regrowth after injury [37,38]. This is due to the size of the reserve of substrate substances in the roots. For example, mono- and disaccharides can account for up to 50% of the total sugar concentration in the roots of *R. crispus* and *R. obtusifolius*. This colossal capacity for vegetative regeneration of *Rumex* plants can be an excellent help in evaluating these plants as potential sources of biologically active substances not only for direct use, but also for biotechnological processing.

Plants of the *Rumex* genus are characterized by the accumulation of a number of biologically active components, such as anthraquinones, naphthalene-1,8-diols, flavonoids, and stilbenoids [7]. Flavonoids are one of the dominant groups of substances that determine the photochemical composition of plants of the genus *Rumex*. The presence of flavan-3-ols and other phenolic compounds in sorrel leaves gives additional advantages to *Rumex* as a raw material rich in physiologically active substances [39]. The flavonoids reported in the *Rumex* species were either flavonols or their O-/C-glycosides. For instance, the apigenin-flavone glucoside vitexin was isolated from *R. acetosa* [40]. The results of our study demonstrate a wide variety of glycosylated quercetin derivatives in experimental plants of *R. acetosa*, including quercetin-3-O-rutinoside (rutin) and quercetin 3-β-D-glucoside (isoquercitrin). Several authors point out the presence of luteolin derivatives in *R. acetosella* plants [41,42], which is also reflected in our results (Table 2). As noted above, species of the subgenus *Rumex* (*R. confertus*, *R. crispus*, *R. maritimus*, *R. obtusifolius*, and *R. sanguineus*) were also characterized by the presence of flavonol derivatives such as kaempferol, whereas they were not found in *R. acetosa* and *R. acetosella*.

In addition to flavonoids, the total pool of phenolic compounds also includes phenolic acids. For example, phenolic acids are widely present in the leaves of *R. acetosa* in relatively high concentrations. Vasas et al. showed that the phenolic acid profile of *R. acetosa* includes trans- and cis-resveratrol (approx. 41 μg g^−1^), vanillic acid (approx. 130 μg g^−1^), and sinapic acid (approx. 5708 μg g^−1^) [7], which is confirmed by our results. In our study, the level of synapic acid in the leaves of *R. acetosa* was 4.9 ± 0.4 mg g^−1^ (4900 μg g^−1^).

The high level of phenolic compounds of plants of the *Rumex* genus largely determines the high radical-inhibiting activity of the extracts. Earlier, in a pharmacological study of *R. crispus* extracts, aqueous extracts of leaves and seeds showed the highest antioxidant activity. In addition, the ethanol extract of *R. crispus* seeds showed a high ability to scavenge the DPPH radical [43,44]. In fact, this is confirmed by our data, according to which *R. crispus* demonstrated the highest values of antioxidant activity in the framework of standard testing (DPPH, FRAP, ABTS) (Table 3). Shagjjav and co-authors demonstrated that extracts of *R. acetosa* have a high level of activity in neutralizing the DPPH radical IC50 1.86 ± 0.06 μg mL^−1^ [45]. It should be noted that the authors point to a high antioxidant activity of extracts of this species, whereas, within the framework of our study, the antioxidant activity of *R. acetosa* was the lowest among all analyzed samples. Available data on the antioxidant activity of *R. acetosella*, *R. confertus*, *R. maritimus*, *R. obtusifolius*, and *R. sanguineus* are rather scarce. Thus, our study can complement the fragmentary picture by using the patterns of the biochemical composition of the *Rumex* wild species.

Flavonoids, which are polyphenols in nature, can play an essential role in the regulation of metabolic processes not only in plant organisms, but also in the organisms that consume them. For example, they can protect cells from destruction, act as anti-inflammatory agents, and participate in redox reactions in cells [39,46]. It is these properties that formed the basis for the widespread use of plants of the genus *Rumex*, not only for traditional medical practices, but also for pharmacological research [47]. For medical purposes, decoctions or infusions are mainly prepared from plant parts [48]. This way, *R. acetosa*, *R. acetosella* (leaf, aerial parts, seeds), *R. crispus* (roots, seeds), and *R. obtusifolius* (aerial parts) are widely used to treat a very wide range of diseases: diarrhea, tumors, ulcers, rashes and wounds, kidney diseases, and ringworm [8,49].

Aerial parts of many species (for example, *R. acetosa*, *R. acetosella*, and *R. crispus*) are widely used for food. Plants are collected mainly in spring and are used as vegetables [6,7,50]. Moreover, the accumulated amount of research allows us to define the plants of this genus as a «superfood». Currently, «superfood» is defined as foods high in nutritional or biologically active phytochemicals beneficial to human health [51]. The results of our studies prove that plants of the genus *Rumex* can occupy a niche in the food industry and act as a functional food product.

## 5. Conclusions

Currently, there is a certain biotechnological demand from the food and pharmacological industries for plants with unique metabolic qualities. The resource base of cultivated agricultural plants is often either limited or not of interest in this aspect. Thus, researchers began to pay more attention to wild flora. Plants of the genus *Rumex* are no exception. This study attempts to optimize and unify data on the content of biologically active substances, as well as data on the antioxidant activity of extracts of the studied species. In addition, the results obtained can serve as an additional argument in the dispute about the distribution of plants of the genus *Rumex* into specific systematic groups. This is especially relevant in the context of the formation of a “new taxonomy”, which is built on the basis of phylogenetic data, which is not always sufficient to formulate unambiguous conclusions.

## Figures and Tables

**Table 1:** Content of some groups of phenolic compounds in the leaves of different *Rumex* species.

Species	TPC ^1^, mg GAE g^–1^	TFC, mg RE g^–1^	THA, mg CAE g^–1^	TCC, mg CE g^–1^	PAs, mg CyE g^–1^	TTC, mg GAE g^–1^
*R. acetosa*	23 ± 2	18 ± 1	12.7 ± 0.6	0.90 ± 0.05	0.24 ± 0.02	0.46 ± 0.05
*R. acetosella*	117 ± 7	106 ± 4	18 ± 1	1.3 ± 0.1	2.2 ± 0.2	11 ± 1
*R. confertus*	76 ± 7	38± 2	4.8 ± 0.3	5.0 ± 0.3	4.0 ± 0.3	6.4 ± 0.3
*R. crispus*	131 ± 10	92± 5	8.9 ± 0.6	5.2 ± 0.3	6.4 ± 0.3	14 ± 1
*R. maritimus*	111 ± 6	120 ± 9	5.8 ± 0.6	4.8 ± 0.3	5.0 ± 0.4	7.1 ± 0.6
*R. obtusifolius*	129 ± 9	92 ± 4	1.9 ± 0.1	6.0 ± 0.4	7.2 ± 0.5	17 ± 1
*R*. *sanguineus*	126 ± 5	99 ± 6	1.9 ± 0.1	10.9 ± 0.6	6.6 ± 0.4	12.9 ± 0.7

^1^ TPC, total phenolics content; TFC, total flavonoids content; THA, total hydroxycinnamic acids; TCC, total catechins content; PAs, total proanthocyanidins content; TTC, total tannins content.

**Table 2:** Content of phenolic acids and flavonoids in the leaves of different *Rumex* species.

Compounds (Retention Time, Min)	Content of Individual Phenolic Compounds, mg g^–1^						
	*R. acetosa*	*R. acetosella*	*R. confertus*	*R. crispus*	*R. maritimus*	*R. obtusifolius*	*R*. *sanguineus*
Flavonoids							
Catechin (9.7)	–	–	–	1.08 ± 0.07	0.17 ± 0.01	1.32 ± 0.07	12.0 ± 0.8
Quercetin 3-O-rutinoside (rutin) (19.3)	3.4 ± 0.2	–	4.3 ± 0.2	10.2 ± 0.7	9.4 ± 0.6	19.0 ± 1.1	8.6 ± 0.5
Quercetin 3-β-D-glucoside (isoquercitrin) (19.9)	0.56 ± 0.03	–	20.2 ± 1.3	31.9 ± 1.8	22.6 ± 1.5	54.8 ± 3.5	49.5 ± 0.3
*Quercetin derivative (16.3)* ^1^	–	–	–	–	39.6 ± 2.9	–	–
*Quercetin derivative (16.9)*	–	–	–	–	27.1 ± 1.5	–	–
*Quercetin derivative (18.3)*	2.4 ± 0.2	–	0.94 ± 0.06	–	–	–	–
*Quercetin derivative (22.73)*	1.31 ± 0.07	–	–	–	–	–	–
*Quercetin derivative (23.1)*	2.0 ± 0.1	–	–	–	–	–	–
*Quercetin derivative (24.1)*	3.4 ± 0.2	–	–	–	–	–	–
Kaempferol 3-O-glucoside (astragalin) (24.7)	*–*	–	1.82 ± 0.09	24.4 ± 1.6	4.4 ± 0.3	5.1 ± 0.3	8.6 ± 0.6
*Kaempferol* *derivative (22.8)*	*–*	–	0.75 ± 0.04	12.9 ± 1.0	1.4 ± 0.1	2.6 ± 0.2	3.2 ± 0.2
*Kaempferol* *derivative (20.9)*	*–*	–	–	–	4.9 ± 0.3	–	–
Luteolin 7-O-glucoside (cynaroside) (20.7)	0.51 ± 0.03	4.3 ± 0.3	–	–	–	–	–
*Luteolin* *derivative (15.5)*	–	89.5 ± 4.7	–	–	–	–	–
*Apigenin* *derivative (19.4)*	–	5.1 ± 0.3					
Phenolic acids							
Gallic acid (3.8)	–	–	–	5.3 ± 0.3	–	0.34 ± 0.02	0.33 ± 0.02
3,4-Dihydroxybenzoic acid (protocatechuic acid) (5.8)	0.12 ± 0.01	0.58 ± 0.03	–	0.56 ± 0.03	0.21 ± 0.01	–	0.21 ± 0.01
Sinapic acid (8.2)	4.9 ± 0.4	1.22 ± 0.08	1.5 ±0.1	–	1.8 ± 0.1	–	–
Caftaric acid (9.2)	1.7 ± 0.1	–	–	–	–	–	–
Chlorogenic acid (10.2)	1.21 ± 0.09	3.04 ± 0.17	1.8 ± 0.1	–	0.19 ± 0.01	–	–
Caffeic acid (10.5)	–	0.93 ± 0.05	–	0.10 ± 0.01	0.29 ± 0.03	–	–
p-Coumaric acid (14.2)	0.15 ± 0.02	–	–	–	–	–	–
Ellagic acid (17.9)	0.28 ± 0.02	–	–	0.83 ± 0.05	–	–	–
*Hydroxybenzoic acid derivative (11.2)*	0.97 ± 0.05	4.0 ± 0.2	–	–	2.9 ± 0.2	–	–
*Hydroxybenzoic acid* *derivative (12.5)*	0.54 ± 0.05	3.0 ± 0.2	–	–	–	–	–

^1^ The compounds identified based on UV spectra and quantified by standard with the same aglycon are indicated in italics.

**Table 3:** Antioxidant activity of extracts from the leaves of different *Rumex* species.

Species	AOA (DPPH) ^1^, mg TE g^–1^	AOA (ABTS), mg TE g^–1^	AOA (FRAP), mg TE g^–1^
*R. acetosa*	3.1 ± 0.3	5.1 ± 0.5	3.9 ± 0.3
*R. acetosella*	31 ± 3	48 ± 3	27 ± 3
*R. confertus*	22 ± 1.3	37 ± 3	39 ± 4
*R. crispus*	69 ± 4	56 ± 4	57 ± 3
*R. maritimus*	31 ± 2	63 ± 4	61 ± 4
*R. obtusifolius*	37 ± 2	48 ± 4	43 ± 2
*R. sanguineus*	35 ± 3	52 ± 4	47 ± 4

^1^ AOA (DPPH), antioxidant activity determined by the DPPH (2,2-diphenyl-1-picrylhydrazyl) assay; AOA (ABTS), antioxidant activity determined by the ABTS (2,2′-azino-bis(3-ethylbenzothiazoline-6-sulfonic acid)) assay; AOA (FRAP), ferric reducing antioxidant power.

**Table 4:** Correlation matrix with the Pearson coefficient values for phenolic compounds and antioxidant activity of *Rumex* extracts.

Variables	TPC ^1^	TFC	THA	TCC	PAs	TTC	DPPH	ABTS	FRAP
TPC	1	0.881 **	–0.317 ^ns^	0.567 *	0.822 **	0.915 **	0.806 **	0.921 **	0.785 **
TFC		1	–0.114 ^ns^	0.368 ^ns^	0.586 *	0.664 *	0.602 *	0.918 **	0.714 **
THA			1	–0.820 **	–0.768 **	–0.354 ^ns^	–0.174 ^ns^	–0.322 ^ns^	–0.563 *
TCC				1	0.809 **	0.537 *	0.389 ^ns^	0.513 *	0.614 *
PAs					1	0.826 **	0.721 **	0.751 **	0.842 **
TTC						1	0.776 **	0.701 **	0.591 *
DPPH							1	0.728 **	0.742 **
ABTS								1	0.909 **
FRAP									1

^1^ TPC, total phenolics content; TFC, total flavonoids content; THA, total hydroxycinnamic acids; TCC, total catechins content; PAs, total proanthocyanidins content; TTC, total tannins content; DPPH, antioxidant activity determined by the DPPH (2,2-diphenyl-1-picrylhydrazyl) assay; ABTS, antioxidant activity determined by the ABTS (2,2′-azino-bis(3-ethylbenzothiazoline-6-sulfonic acid)) assay; FRAP, ferric reducing antioxidant power. ** Correlation is significant at *p* ≤ 0.01; * correlation is significant at *p* ≤ 0.05; ^ns^, correlation is not significant (*p* > 0.05).

## Data Availability

Not applicable.
